# Supplementary material for: Strong-coupling and high-bandwidth cavity electro-optic modulation for advanced pulse-comb synthesis
Source: Light Sci Appl. 2025 Oct 22;14:373. doi: 10.1038/s41377-025-02046-y (PMC12546911; doi:10.1038/s41377-025-02046-y)
Supplement: Supplementary file 1 — Supplementary Information for: Strong-coupling and high-bandwidth cavity electro-optic modulation for advanced pulse-comb synthesis [file 41377_2025_2046_MOESM1_ESM.docx]

**Supplementary Information for: Strong-coupling and high-bandwidth cavity electro-optic modulation for advanced pulse-comb synthesis**

Tianqi Lei^1,*^, Yunxiang Song^2,3,*^, Yanyun Xue^1^, Qihuang Gong^1,4^, Marko Lončar^2,†^, Yaowen Hu^1,4,†^

^1^State Key Laboratory for Mesoscopic Physics and Frontiers Science Center for Nano-optoelectronics, School of Physics, Peking University, Beijing 100871, China

^2^John A. Paulson School of Engineering and Applied Sciences, Harvard University, Cambridge, MA 02138, USA

^3^Quantum Science and Engineering, Harvard University, Cambridge, MA 02138, USA

^4^Collaborative Innovation Center of Extreme Optics, Shanxi University, Taiyuan, China.

^*^These authors contributed equally.

^†^Corresponding authors: [loncar@seas.harvard.edu](mailto:loncar@seas.harvard.edu); [yaowenhu@pku.edu.cn](mailto:yaowenhu@pku.edu.cn)

**Table of Contents**

[Theoretical derivation of the electro-optic Hamiltonian for arbitrary bandwidth and strength modulation 2](#_Theoretical_derivation_of)

[Band structure calculations for electro-optic Hamiltonian with arbitrary bandwidth and strength modulation](#_Band_structure_calculations) [5](file:///C:\Users\ltqpe\Downloads\universal_EOC_SI_v5_TL_YH.docx#_Toc184744996)

[Variation of electro-optic modulation with modulation depth under zero pump detuning 5](#_Variation_of_electro-optic)

[Phase transition from pump insulating regime to electro-optic pulse excitation under maximum pump detuning 6](#_Phase_transition_from)

[Correspondence between band structure and modulation waveform 7](#_Correspondence_between_band)

[Process of machine learning based flat comb generation 7](#_Process_of_machine)

[Frequency boundary reflection effects in the machine learning process at high modulation bandwidth 8](#_Frequency_boundary_reflection)

[Microwave detuning in machine learning optimization 9](#_Microwave_detuning_in)

[Microwave detuning for creating artificial frequency boundaries to control flat comb width 10](#_Microwave_detuning_for)

[Supplementary references 11](file:///C:\Users\ltqpe\Downloads\universal_EOC_SI_v5_TL_YH.docx#_Toc184745006)

Supplemetary figures S1-S12 and table S1.

Supplemetary videos.

# **Theoretical derivation of the electro-optic Hamiltonian for arbitrary bandwidth and strength modulation.**

We start from the nonlinear Maxwell equations as

$$\nabla\times H=\frac{\partial D}{\partial t}$$

$$\nabla\times E=-\frac{\partial B}{\partial t}$$

$$\nabla\cdot B=0$$

$$\nabla\cdot D=0$$

Where

$$D=\varepsilon\varepsilon_{0}E+P_{\mathrm{NL}}$$

$$B=\mu\mu_{0}H$$

and $P_{NL}$is the nonlinear susceptibility. We then derive the Helmholtz equation with nonlinear terms as

$$\nabla^{2}E-\frac{n^{2}}{c^{2}}\frac{\partial^{2}E}{\partial t^{2}}=\mu_{0}\frac{\partial^{2}}{\partial t^{2}}P_{\mathrm{NL}}$$

We expand the electric field into the following form as

$$E=\sum_{n} E_{n}(z,t)E_{n\perp}(x,y)e^{ik_{nz}z-i\omega_{n}t}$$

In the next derivation, we only consider the change in electric field propagation $E_{n}(z,t)$ and not the transverse distribution of the electric field $E_{n\perp}(x,y)$. This is reasonable as we just need to separate the variables in the Helmholtz equation above. For convenience, we will eliminate the phase propagation term $e^{ik_{n}z}$ in the subsequent derivation and assume the phase matching, so it is reasonable to eliminate the phase propagation term. The LHS of the Helmholtz equation can now be written as

$$\sum_{n} {\frac{\partial^{2}}{\partial z^{2}}E}_{n}\left( z,t \right)+2ik_{n}\frac{\partial}{\partial z}E_{n}-k_{n}^{2}E_{n}+\frac{n^{2}\omega_{n}^{2}}{c^{2}}E_{n}\approx\sum_{n} 2ik_{n}\frac{\partial}{\partial z}E_{n}e^{ik_{n}z-i\omega_{n}t}$$

In this process, we use the slowly varying amplitude approximation, that is, we assume that ${\frac{\partial^{2}}{\partial z^{2}}E}_{n}\left( z,t \right)\ll2ik_{n}\frac{\partial}{\partial z}E_{n}$. Next, we analyze the RHS of the Helmholtz equation.

$$P_{\mathrm{NL}}=\varepsilon_{0}\chi^{2}E^{0}E=\varepsilon_{0}\Delta\varepsilon\left( z,t \right)E=\varepsilon_{0}\Delta\varepsilon\left( z,t \right)\sum_{n} E_{n}(z,t)e^{ik_{n}z-i\omega_{n}t}$$

$$\frac{\partial^{2}}{\partial t^{2}}P_{\mathrm{NL}}=\varepsilon_{0}\left( \frac{\partial^{2}}{\partial t^{2}}\Delta\varepsilon\left( z,t \right)\sum_{n} E_{n}\left( z,t \right)+2\frac{\partial}{\partial t}\Delta\varepsilon\sum_{n} \frac{\partial}{\partial t}E_{n}\left( z,t \right)+2\frac{\partial}{\partial t}\Delta\varepsilon\sum_{n} E_{n}\left( z,t \right)(-i\omega_{n})+2\Delta\varepsilon\sum_{n} \frac{\partial}{\partial t}E_{n}\left( z,t \right)(-i\omega_{n})+\Delta\varepsilon\left( z,t \right)\sum_{n} \frac{\partial^{2}}{\partial t^{2}}E_{n}\left( z,t \right)+\Delta\varepsilon\sum_{n} E_{n}\left( z,t \right){(-i\omega_{n})}^{2} \right)$$

$E^{0}$ denotes the electric field amplitude of the microwave modulation. We can simplify the above equation by slow varying envelope approximation (SVEA). Since the optical frequency term is much larger than other frequencies in the system, we only keep the last term in the above equation.

$$\sum_{n} 2ik_{n}\frac{\partial}{\partial z}E_{n}e^{-i\omega_{n}t}=-\Delta\varepsilon\sum_{n} \frac{{\omega_{n}}^{2}}{c^{2}}E_{n}\left( z,t \right)e^{-i\omega_{n}t}$$

We have two solutions for the above formula. The first is to write the specific form of $\Delta\varepsilon=\chi^{2}E^{0}cos\omega t$, and we get the formula as

$$\frac{\partial}{\partial z}E_{n}=\frac{i\Delta\varepsilon k}{2n^{2}}(E_{n-1}\left( z,t \right)+E_{n+1}\left( z,t \right))$$

By integrating the modulation electrode area, we can get the electric field after phase modulation. The second solution is to directly integrate the above equation over the modulation electrode region.

$$\int dz\sum_{n} 2ik_{n}\frac{\partial}{\partial z}E_{n}e^{-i\omega_{n}t}=\int dz(-\Delta\varepsilon\sum_{n} \frac{{\omega_{n}}^{2}}{c^{2}}E_{n}\left( z,t \right)e^{-i\omega_{n}t})$$

Then we swap the order of integration and summation as

$$\sum_{n} e^{-i\omega_{n}t}\int dz2ik_{n}\frac{\partial}{\partial z}E_{n}=\sum_{n} e^{-i\omega_{n}t}\int dz\frac{-\Delta\varepsilon{\omega_{n}}^{2}}{c^{2}}E_{n}\left( z,t \right)$$

In order for the equation to hold true, we need

$$\int dz2ik_{n}\frac{\partial}{\partial z}E_{n}=\int dz\frac{-\Delta\varepsilon{\omega_{n}}^{2}}{c^{2}}E_{n}\left( z,t \right)$$

The integral of the above formula is very simple, namely

$$E_{n}\left( z+L,t \right)=exp\left( i\frac{\Delta\varepsilon(t)kL}{2n^{2}} \right)E_{n}\left( z,t \right)=exp\left( i\beta(t) \right)E_{n}\left( z,t \right)$$

We find that the final effects of these two solutions are exactly the same. In fact, the two solutions actually treat electro-optic modulation from two different perspectives. The first is that electro-optic modulation constructs coupling between adjacent frequencies. The second is that electro-optic phase modulation modulates the input electric field, i.e. the electro-optic modulator is equivalent to a black box, which produces a transmittance $T=e^{i\beta(t)}$ for the incident light field. The output light field of the black box is

$$E_{\mathrm{out}}=TE_{\mathrm{in}}=e^{i\beta(t)}E_{\mathrm{in}}$$

This black box approach allows us to ignore details such as the electro-optic modulation structure parameters and simplifies the following discussion.

Next, we consider the effect of the microcavity on the light field. Notably, unlike a straight waveguide, the cavity EO system mandates a time scale corresponding to the round-trip time $T_{R}$(or its integer multiples) for light circulating within the microcavity. We study the cavity electro-optic modulator using an equivalent black-box transmittance and derive the effective Hamiltonian on a discrete-time scale. Subsequently, we discuss the necessity of employing discrete-time coupled mode equations within the cavity EO modulation system, and finally, we present the discrete-time coupled mode equations derived from the effective Hamiltonian for use in our numerical computations.

Considering the light field at the coupling point between the waveguide and the microcavity, we analyze the effect of the cavity EO system with electro-optic modulation on the light field as it circulates around the microcavity. The modulation effect is embodied in the Hamiltonian, and to incorporate it, we simply quantize the light field. Inspired by the approach in^1^ and illustrated in Fig. S1, we decompose the effect of the cavity EO system on the light field at time 𝑇 into two parts: the scattering and loss of the cavity, yielding a transmittance $T_{\mathrm{loss}}=e^{-\gamma}$ $\gamma$represents the cavity loss), and the phase modulation, imparting a transmittance $T_{\mathrm{phase}}=e^{i\beta(t)}$. Consequently, after one round trip $T_{R}$around the microcavity, the light field satisfies $E\left( t+t_{R} \right)=E\left( t \right)\times T_{\mathrm{loss}}\times T_{\mathrm{phase}}+\sqrt{k}E_{\mathrm{in}}$, where $E_{\mathrm{in}}$ denotes the pump coupled into the microcavity and k is the coupling strength between the microcavity and the waveguide. To derive the discrete-time coupled mode equations, we define the discrete-time ($T=t_{R},2t_{R}\ldots$) differential operator $\frac{\partial}{\partial T}$

$$\frac{\partial E\left( t \right)}{\partial T}:=\frac{E\left( t+t_{R} \right)-E(t)}{t_{R}}$$

Substituting $E\left( t+T_{R} \right)$ into the above formula, we can get

$$t_{R}\frac{\partial E\left( t \right)}{\partial T}=\left( T_{\mathrm{total}}-1 \right)E\left( t \right)+\sqrt{\kappa_{e}}\sqrt{P_{\mathrm{in}}}$$

Where $T_{\mathrm{total}}=T_{\mathrm{loss}}\times T_{\mathrm{phase}}, P_{\mathrm{in}}=f_{R}E_{\mathrm{in}}^{2}$, quantizing the light field in the above equation, we get the Heisenberg equation

$$\frac{\partial a}{\partial T}=-f_{R}\left( 1-T_{\mathrm{total}} \right)a+\sqrt{\kappa_{e}}\sqrt{P_{\mathrm{in}}}$$

And finally, we get the Hamiltonian

$$H=-if_{R}(1-T_{\mathrm{total}})a^{\dagger}a$$

We assumed $\hbar=1$. In the regime where $\beta\ll1$, we can employ the following small-signal approximation: $e^{-\gamma}e^{i\beta sin(\omega t)}=1-\gamma+i\beta sin(\omega t)$. This linearization considerably simplifies the analysis of the system's dynamics while remaining accurate for sufficiently small modulation depths.

$$H=-if_{R}\left( 1-\left( 1-\gamma+i\beta\sin\left( \omega t \right) \right) \right)a^{\dagger}a=-i\kappa a^{\dagger}a-\Omega sin(\omega t)a^{\dagger}a$$

The term $\Omega\sin\left( \omega t \right)a^{\dagger}a$ is widely employed in cavity electro-optic modulation systems, representing the small-signal approximation of the effective Hamiltonian in the strong modulation regime. To gain deeper insight into the complex dynamical effects induced by strong electro-optic modulation, we neglect losses and retain only the coupling terms, thereby deriving the final interaction Hamiltonian.

$$H_{\mathrm{int}}=i f_{R} e^{i\beta\left( t \right)}(\sum_{n} \sum_{m} a_{n+m}^{\dagger}a_{n}+h.c.)$$

Therefore, for the cavity EO system, even for single-tone modulation, its Hamiltonian brings about interactions between non-neighboring frequency lattices. Analyzing from the perspective of the monitoring port helps us understand this Hamiltonians. Considering that we are observing at the output end of the coupled waveguide in Fig. S1, first of all, we cannot monitor the light field at each position in the microcavity in real time through the waveguide at the output end. We can only monitor the light field at the coupling point between the waveguide and the microcavity. Secondly, the effect of the cavity EO system on the light field is not instantaneous. The time scale of the interaction is $t_{R}$. The light coupled into the microcavity at time $t$ will be output at $t+t_{R}$. Finally, for the microcavity, due to the existence of the electro-optic modulator, the propagation of the electro-optic pulse in the microcavity cannot always remain steady state. Here, we point out that within one roundtrip time $t_{R}$, several physical processes take place: pump coupling and coupling loss, cavity scattering and absorption losses in the cavity, and sideband generation through microwave modulation by the cavity electro-optic modulator. When the timescale under consideration is shorter than $t_{R}$, these processes can no longer be approximated as a steady and unified pump-dissipation mechanism. Traditional Hamiltonian formulations typically neglect this timescale mismatch, and the resulting coupled-mode equations fail to yield valid steady-state solutions in the strong-coupling regime of cavity electro-optic modulation. The electro-optic pulse can only remain steady when the time scale considered is an integer multiple of $t_{R}$. The transmission curves of the electro-optic modulation of the straight waveguide and the microcavity electro-optic modulation are drawn as showed in Fig. S2. The straight waveguide will not produce electro-optic pulses because the phase modulation will not affect the amplitude, but the microcavity can produce electro-optic pulses with a period of $t_{R}$. In summary, cavity EO systems operate on the electro-optic pulse, and the time scale of this operation cannot be regarded as infinitesimal.

Building on the above discussion, we will further demonstrate that, owing to the integral over each roundtrip time, the non-Hermitian properties of the effective Hamiltonian on this discrete-time scale remains fully compatible with energy conservation. To verify that we calculated the effective Hamiltonian expectation value at two adjacent discrete time $t$ and $t+t_{R}$.

$${<H}_{\mathrm{int}}>\left( t+t_{R} \right)=<\psi\left( t+t_{R} \right)\left| H_{\mathrm{int}}\left( t+t_{R} \right) \right|\psi\left( t+t_{R} \right)>$$

While

$$|\psi\left( t+t_{R} \right)>=|\psi\left( t \right)>-it_{R}H_{\mathrm{int}}\left( t \right)|\psi(t)>$$

So the expectation can be written as

$${<H}_{\mathrm{int}}>\left( t+t_{R} \right)=(<\psi\left( t \right)\left| +<\psi(t)|it_{R}{H_{\mathrm{int}}}^{\dagger}\left( t \right))H_{\mathrm{int}}\left( t+t_{R} \right)( \right|\psi\left( t \right)>-it_{R}H_{\mathrm{int}}\left( t \right)|\psi(t)>)$$

Due to the periodicity of the effective Hamiltonian

$$H_{\mathrm{int}}\left( t+t_{R} \right)=i f_{R} (e^{i\beta cos\left( \omega(t+t_{R}) \right)}-1)(\sum_{n} \sum_{m} a_{n+m}^{\dagger}a_{n}+h.c.)$$

$$H_{\mathrm{int}}\left( t+t_{R} \right)=H_{\mathrm{int}}\left( t \right)$$

We get

$${<H}_{\mathrm{int}}>\left( t+t_{R} \right)={<H}_{\mathrm{int}}>\left( t \right)+<\psi\left( t \right)\left| (it_{R}{H_{\mathrm{int}}}^{\dagger}\left( t \right)-it_{R}H_{\mathrm{int}}\left( t \right)+t_{R}^{2}{H_{\mathrm{int}}}^{\dagger}\left( t \right)H_{\mathrm{int}}\left( t \right))H_{\mathrm{int}}\left( t \right) \right|\psi\left( t \right)>$$

By substituting the provided $H_{int}$ into the above expression, one immediately sees that the second term on the right-hand side vanishes. Therefore, ${<H}_{\mathrm{int}}>\left( t+t_{R} \right)={<H}_{\mathrm{int}}>\left( t \right)$. As a result, we confirm the energy conservation on the discrete-time scale of our effective Hamiltonian.

Following the preceding discussion, we perform our analysis of the cavity electro-optic modulation system on the discrete time scale $t_{R}$. For single-tone modulation, the microwave phase proves to be inconsequential to the dynamics of the cavity electro-optic system. Consequently, without loss of generality, we adopt a sinusoidal microwave modulation and employ the Bessel function expansion in our analysis.

$$e^{i\beta cos(\omega t)}=\sum J_{m}\left( \beta\right)i^{m}e^{im\omega t}$$

By incorporating the effective Hamiltonian and employing the rotating-wave approximation (RWA), we derived the coupled mode equations on a discrete time scale under single-tone modulation condition

$$\frac{\partial a_{n}}{\partial T}=f_{R}\left( \left( 1-\gamma\right)J_{0}\left( \beta\right)-1 \right)a_{n}+f_{R}\left( 1-\gamma\right)\sum_{m\neq0} i^{m}J_{m}\left( \beta\right)a_{n+m}+\sqrt{\kappa_{e}}\sqrt{P_{\mathrm{in}}}\delta_{n,0}$$

Usually we set $\gamma\ll1$

$$\frac{\partial a_{n}}{\partial T}=\left( f_{R}\left( J_{0}\left( \beta\right)-1 \right)-\frac{\kappa}{2} \right)a_{n}+f_{R}\sum_{m\neq0} i^{m}J_{m}\left( \beta\right)a_{n+m}+\sqrt{\kappa_{e}}\sqrt{P_{\mathrm{in}}}\delta_{n,0}$$

In the presence of pump detuning, an additional phase accumulation $e^{i\varphi_{0}}$ occurs, where $\varphi_{0}=(\omega-\omega_{0})t_{R}$. Here, $\omega_{0}$denotes the angular frequency of the cavity resonance mode that is nearest to the pump light $\omega$. Given that $e^{i\varphi_{0}}$ may not be infinitesimal under conditions of substantial detuning, we refrain from approximating the detuning term, thereby obtaining the coupled mode equations under detuned conditions.

$$\frac{\partial a_{n}}{\partial T}=f_{R}\left( \left( 1-\gamma\right)e^{i\varphi_{0}}J_{0}\left( \beta\right)-1 \right)a_{n}+f_{R}\left( 1-\gamma\right)e^{i\varphi_{0}}\sum_{m\neq0} i^{m}J_{m}\left( \beta\right)a_{n+m}+\sqrt{\kappa_{e}}\sqrt{P_{\mathrm{in}}}\delta_{n,0}$$

For an arbitrary modulation waveform, the Bessel expansion is no longer applicable. Instead, by substituting a Fourier transform approach $\mathcal{F}\left\{ e^{i\beta(t)} \right\}\left( \omega\right)$, we derived the cavity electro-optic modulation coupled mode equations that remain valid under any modulation waveform, modulation strength, and laser detuning conditions.

$$\frac{\partial a_{n}}{\partial T}=f_{R}\left( \left( 1-\gamma\right)e^{i\varphi_{0}}\mathcal{F}\left\{ e^{i\beta(t)} \right\}\left( 0 \right)-1 \right)a_{n}+f_{R}\left( 1-\gamma\right)e^{i\varphi_{0}}\sum_{m\neq0} i^{m}\mathcal{F}\left\{ e^{i\beta(t)} \right\}\left( m\omega_{m} \right)a_{n+m}+\sqrt{\kappa_{e}}\sqrt{P_{\mathrm{in}}}\delta_{n,0}$$

For the multi-wavelength pumping scenario, the pump term in the coupled mode equations must be reformulated as a vector $(\sum_{m} \sqrt{P_{\mathrm{in}}}\delta_{n,m})$.

For the dispersion effect, we can add additional dispersion term in the coupled mode equations as

$$\frac{\partial a_{n}}{\partial T}=(f_{R}\left( \left( 1-\gamma\right)e^{i\varphi_{0}}\mathcal{F}\left\{ e^{i\beta\left( t \right)} \right\}\left( 0 \right)-1 \right)+iD_{int}\left( n \right))a_{n}+f_{R}\left( 1-\gamma\right)e^{i\varphi_{0}}\sum_{m\neq0} i^{m}\mathcal{F}\left\{ e^{i\beta(t)} \right\}\left( m\omega_{m} \right)a_{n+m}+\sqrt{\kappa_{e}}\sqrt{P_{\mathrm{in}}}\delta_{n,0}$$

Where $D_{int}\left( n \right)= \omega_{n}-\omega_{0}-nFSR=\sum_{m\geq2} \frac{D_{m}}{m!}n^{m}$ is the integrated dispersion.

To solve the Hamiltonian-based coupled modes equations numerically, we must truncate the number of coupled modes 𝑁 included in the numerical solution and the maximum order 𝑚 of high-order coupling induced by the microwave modulation. Because of the Bessel functions $J_{m\geq m_{0}}(\beta)\to0$ when *m* is larger than the truncating point $m_{0}$ as shown in Fig. S3a. We can choose the suitable truncating point mathematically justified as we plot in Fig. S3b.

# **Band structure calculations for electro-optic Hamiltonian with arbitrary bandwidth and strength modulation.**

The synthesized frequency dimension of EO modulation can be analogized to solid-state physics. Just as the periodic arrangement of atoms in real space forms energy bands in reciprocal space (the Brillouin zone), the periodic arrangement of cavity resonance frequencies in the frequency dimension also leads to energy bands^2^. Interestingly, the reciprocal space of the frequency dimension corresponds to $\frac{1}{f}=t$ in the time dimension. This allows us to calculate time-domain pulses shape under different pump detuning corresponding to the energy band structure of the cavity EO system. This provides a convenient method for measuring the energy bands of the cavity EO system and enhances our understanding of it. Specifically, the cavity EO system provides a series of evenly spaced optical modes in the frequency dimension, denoted as $a_{m}$. These modes can be expanded as Bloch vectors $a_{m}=\int dke^{im\omega k}a\left( k \right)$, with the inverse transformation given by $a\left( k \right)=\sum a_{m}e^{-im\omega k}$. Since the periodicity of optical modes’ reciprocal space is $\frac{1}{f}=t$ in the time domain. Thus, we can substitute $k\to t$, yielding $a\left( k=t \right)=\sum a_{m}e^{-im\omega t}$, which represents the EO modulation time-domain pulse. This shows that the time-domain EO pulse reflects the energy band structure of the cavity EO system.

# **Variation of electro-optic modulation with modulation depth under zero pump detuning.**

In this section, to demonstrate the transition of EO modulation from the weak-coupling regime to the strong-coupling regime, we gradually increase the EO modulation strength $\Omega=\frac{\beta}{2\pi}\omega_{R}$ from 0 to multiple FSRs. We calculate the EO spectra by varying the modulation strength from 0 to multiple FSRs as shown in Fig. S4, using the Hamiltonian above. When $\Omega<\omega_{R}$, the EO comb shape remains unchanged, and its slope satisfies $k\sim\frac{\beta}{Q}$, where $\beta$ is the modulation depth and $Q$ is the cavity quality factor. $\beta$ characterizes the coupling strength between adjacent frequency lattice points, while $Q$ determines the loss of pump energy as it passes through the frequency lattice. This indicates that at $\Omega<\omega_{R}$ the nearest-neighbor coupling dominates in the weak-coupling regime. i.e. Pump energy propagates step by step along the frequency lattice. The spectra shape change happens when $\Omega$ a little less than FSR, due to the linewidth of the cavity resonance peaks. When $\Omega>\omega_{R}$, the system undergoes a phase transition from weak coupling to strong coupling. The spectra begin to display a periodic oscillation envelope, indicating the emergence of long-range coupling interactions in the EO modulation system. As Ω exceeds more FSRs, new small peaks appear between the periodic envelopes, the period of the envelope changes too, signifying the presence of even longer-range interactions.

Then we perform Fourier transform on the calculated spectra to obtain the time-domain EO pulses in Fig. S5. The Fourier transform results are consistent with the cavity resonance-modulated results discussed in the paper, demonstrating the transition from the weak to the strong coupling region. In the weak-coupling regime, we get two pulses in one roundtrip as the EO modulation causes the cavity resonance sweeping across the pump laser twice in one roundtrip, generating two EO modulated pulses. As the modulation strength increases, the number of EO pulses remains constant, but the pulse width narrows, because we differentiate the EO modulation waveform to obtain the slope, which corresponds to the cavity resonance peak sweeping speed across the pump laser. Therefore, the rising and falling time of the modulation waveform correspond to the pulse width. Here, due to the symmetry of single-tone modulation, the two pulses have the same pulse width and narrow at the same speed. We know the narrower time domain pulse means the wider spectra, corresponding to $k\sim\frac{\beta}{Q}$ in previous paragraph. When $\Omega>\omega_{R}$, the system enters the strong coupling region, and the number of EO pulses in the cavity undergoes sudden change. This is because the neighbor energy levels of the cavity pump mode can also be excited within one roundtrip, inducing complex long-range interactions between the cavity frequency lattice. We also show the evolution diagram of EO pulses with modulation strength Ω in Fig. S6. In summary, all of the data and analysis above aligns with our EO modulation theory in the main text, confirming the consistency of our theory.

# **Phase transition from pump insulating regime to electro-optic pulse excitation under maximum pump detuning.**

In this section, we present the theoretical calculation of EO modulation under the maximum pump detuning condition. We demonstrate the system phase transition from the pump insulation region to the weak and strong coupling region in Fig. S7. We keep the laser pump detuning to the maximum detuning $\Delta=\omega_{R}/2$, and gradually increase the EO modulation strength $\Omega$, just as what we do in previous paragraph. However, unlike the case of zero detuning, in the initial stage, no EO pulses are excited. The system is actually insulation to the pump, meaning that the pump energy cannot flow through the cavity resonance frequency lattice. This can also be reflected in the band diagram, where there is a forbidden gap between adjacent energy levels, and the pump with maximum detuning is exactly within this forbidden gap, hence it is insulated.

As $\Omega$ increases, specifically $\Omega>\omega_{R}/2$, EO pulses are excited as show in Fig. S8, indicating a phase transition from the insulating state to a conducting state. This transition can also be clearly reflected in the band diagram, where the modulation strength increasement leads to overlap of energy bands, allowing any detuned laser exciting the EO comb. This brings an intriguing result that under this condition, the cavity EO system is robust to any pump frequency, not observed in other nonlinear microcavity systems such as those utilizing the Kerr effect. As the Fig. S8 shows, the EO pulse number increases from 0 to 2, exhibiting similar behavior to the zero-detuning case. As the modulation strength is further increased, the EO pulse number increases more and enters the strong coupling region.

To show more information of this EO modulation phase transition, we also calculate the conversion efficiency of the EO comb $\eta$, as shown in Fig. S9 a. Initially, the conversion efficiency decreases as $\Omega$ increases. This is because the EO modulation sweeps the pump laser faster, reducing the time during which the pump laser can effectively couple into the cavity. When the modulation strength exceeds the FSR, the $\eta$ suddenly increases because the EO pulses number increasement causes more pump laser coupled into the cavity in one roundtrip. The process then repeats: the conversion efficiency decreases and then increases suddenly when $\Omega$ exceeds the integer of FSRs. In summary, all of the data and analysis above aligns with our EO modulation theory in the main text, confirming the consistency of our theory.

# **Correspondence between band structure and modulation waveform.**

In this section, we provide a detail analysis of the correspondence between the band structure and the modulation waveform. As mentioned earlier, the band structure is essentially the superposition of time-domain EO pulses of different pump detuning. These EO pulses originate from the modulation waveform, With the pump detuning performing as a bias. The combination of modulation waveform and detuning determines the pulses position and width. Additionally, the modulation strength determines the amplitude of system band structure. Therefore, the resulting band structure has a one-to-one correspondence with the modulation waveform. The discussion above doesn’t focus on the specific shape of modulation waveform, so it also works on arbitrary modulation waveform. Building on this principle, we can readily achieve arbitrary band control of EO modulation and explore the topological features of the synthetic frequency domain.

# **Process of machine learning based flat comb generation.**

Our theory supports EO modulation with arbitrary modulation strength and bandwidth, allowing for numerous degrees of freedom provided by different modulation frequencies, strengths, and phases to facilitate arbitrary spectra shape generation. As discussed earlier, we outlined the cavity EO modulation Hamiltonian. We propose that generating EO spectra with arbitrary shapes essentially involves constructing a unique cavity EO modulation Hamiltonian. Here, it is crucial to distinguish between the forward and inverse problems. The forward problem involves calculating the EO spectra shape given by the specific Hamiltonian, whereas the inverse problem requires finding the best Hamiltonian based on a target spectra shape. For the inverse problem, machine learning is the most suitable approach. Before applying machine learning to generate arbitrary spectra shape combs, the following steps are necessary. First, define the machine learning effective parameters range and set a target spectrum shape. Second, establish a loss function to evaluate the effect of machine learning. By adjusting the coefficients of the loss function, we can control the machine learning parameter such as learning regime towards to the target spectra shape. Third, the initial modulation Hamiltonian (usually a single-tone modulation, but it can also start from any modulation form such as a purely random state) is input into the EO comb solver to generate corresponding spectrum. Fourth, the generated spectrum is then sent to the error solver to calculate the error with target shape and derive the loss function. Based on the loss function, the modulation Hamiltonian is updated iteratively until the loss falls below a predefined threshold or the training process reaches a preset number of epochs. Fifth, the machine learning unit system outputs both the optimized EO modulation waveform by the EO modulation waveform reconstructor and the corresponding generated spectrum shape by the EO comb solver. To demonstrate this progress in more detail, we visualize the machine learning progress in Fig. S12 and supplement video. The preset parameter of the EO cavity system is $\mathrm{FSR}\sim3.25 \mathrm{GHz}$, $\kappa_{e}=\kappa_{i}\sim120 \mathrm{MHz}$ , the modulation bandwidth is set as $\mathrm{Bandwidth}\leq9\times\mathrm{FSR}<30 \mathrm{GHz}$ so it can be experimentally achieved. We also add microwave driving detuning as $\delta=22 \mathrm{MHz}$ to help flatten the EO spectra (more detailed discussion later). Here we point out that due to physical constraints and losses inherent in the system, machine learning may fail in generating arbitrary spectra shapes. See more machine learning numerical calculation details in next section. The trained modulation waveform is showed as $\beta\left( t \right)=\sum_{n=1,9} \beta_{n}cos(\omega_{n}t+\phi_{n})$ in table S1.

# **Frequency boundary reflection effects in the machine learning process at high modulation bandwidth.**

In numerical simulations, we typically consider a finite number of modes, ranging from $-N$ to $+N$, where the 0 mode represents the pump mode. This approach essentially truncates the frequency modes, serving as an approximation of the infinitely extended frequency modes. Such an approximation is generally justified for two reasons: first, modes far from the pump mode carry negligible power, rendering them undetectable. Second, the dispersion of cavity modes inhibits the transmission of pump power to the infinite mode far away from pump mode.

Nevertheless, it is essential to consider the boundary reflection effect introduced by this approximation. Truncating the periodic frequency lattice artificially imposes a "hard boundary" in the frequency domain as shown in Fig. S11 b. This phenomenon has been extensively studied in the literature before^3–5^. It can be understood as follows: pump energy flows along the frequency lattice and encounters an abrupt hard boundary, the boundary reflects the energy flow. The interference between the reflected and incident energy results in spectral oscillations near the boundary, akin to a standing wave effect. This boundary reflection causes "distortion" in the numerical spectrum compared to the actual spectrum, as no such artificial ‘hard boundary’ exists in the real frequency lattice.

This effect is generally mild as the reflected energy flow is attenuated during propagation through the lattice, typically affecting only a few modes near the boundary. As illustrated in Fig. S10, excluding the distorted boundary reflection regions, the spectrum elsewhere remains undistorted. However, when the modulation bandwidth starts to increase more than one FSR, the situation becomes vastly different. The boundary-induced reflection effect faces significant challenges in numerical simulations at high modulation bandwidths. If left unaccounted for this effect in the machine learning process, it can lead to substantial spectral distortion, ultimately causing the failure of machine learning. The underlying reason is that reflected energy flows propagate faster and decay more slowly along the fast channels induced by high-frequency modulation, thereby affecting a great number of modes. For instance, under frequency modulation of one FSR, boundary effects affect 20 modes, whereas under frequency modulation of ten FSRs boundary effects influence over 200 modes, resulting in strong distortions in numerical simulations during the machine learning process. Even higher modulation frequencies can impact thousands of modes. More critically, as more modulation frequencies are introduced, the boundary reflection effects compound, creating increasingly complex distortions. It becomes nearly impossible to directly compensate for these distortions under complex high-bandwidth modulation conditions. This challenge resulted in the failure of early machine learning attempts to generate arbitrarily shaped spectra.

# **Microwave detuning in machine learning optimization.**

To mitigate the boundary reflection, particularly the severe distortion under high modulation bandwidth conditions, one solution is to increase the number of modes considered during computation. However, this strategy directly enlarges the size of matrices involved in machine learning and requires prohibitively large computations.

This solution behaves badly because it increases the size of the matrix by $O(N^{2})$ and increases the total machine learning time by $O(N^{3})$. For example, when setting the modulation bandwidth as 10 × FSR, to avoid the distortion influence the result of machine learning, the number of modes must be increased by a factor of 10, leading to a 1000-fold increase in time—impractical for most machine learning processes. As a result, it is very necessary to find an alternative method to eliminate the boundary reflection during the machine learning process without significantly increasing computational demands. Actually, the shape around the frequency boundary is not of primary concern, rather, we are focused on preventing boundary reflections from affecting the region near the pump mode, where accurate results are essential for generating arbitrarily shaped spectra. Instead, our approach leverages physical principles to mitigate the boundary reflection effects, allowing the spectrum computed to remain accurate without increasing matrix size and, consequently, without excessive computational overhead.

As illustrated in Fig. S11 a, the method involves introducing detuning in microwaves. This detuning causes the frequency lattice to slowly deviate from the resonant modes of the microcavity. Because this process is gradual, the reflection effect is relatively weak. This can be analogy as a ‘soft boundary’ as showed in Fig. S11 c. The artificial truncation of the frequency modes induced ‘hard boundary’ exhibits like a hard wall against the power flow from the pump mode, which causes the spectrum severe oscillation, while the microwave detuning induced ‘soft boundary’ can significantly mitigate this oscillation

The residual weak oscillations can be mitigated through further machine learning optimization, thus truly combining the advantages of machine learning and microwave detuning to generate a flat comb spectrum. We discover that introducing microwave detuning significantly improves the flatness of the comb spectrum. From an energy conservation perspective, the detuning creates a boundary that restricts pump energy from propagating beyond the region of interest. This localization of pump energy within the flat comb range enhances its overall flatness.

# **Microwave detuning for creating artificial frequency boundaries to control flat comb width.**

Adjusting the microwave detuning allows the frequency to deviate from the microcavity's resonant modes at a controllable rate, thereby controlling the position of the frequency boundary and enabling control over the flat comb's bandwidth, achieving a programmable-width flat comb. As a result, we don’t need to train again but just adjust the microwave detuning to control the flat comb width. The position of the microwave detuning induced frequency boundary is approximately inversely proportional to detuning $\mathrm{Modenumber}_{\mathrm{boundary}}\propto\frac{\omega_{R}}{\delta}$.

**Supplementary references**

1. Wang, K. *et al.* Generating arbitrary topological windings of a non-Hermitian band. *Science* **371**, 1240–1245 (2021).

2. Dutt, A. *et al.* Experimental band structure spectroscopy along a synthetic dimension. *Nat. Commun.* **10**, 3122 (2019).

3. Dutt, A. *et al.* Creating boundaries along a synthetic frequency dimension. *Nat. Commun.* **13**, 3377 (2022).

4. Hu, Y. *et al.* Mirror-induced reflection in the frequency domain. *Nat. Commun.* **13**, 6293 (2022).

5. Buscaino, B., Zhang, M., Loncar, M. & Kahn, J. M. Design of Efficient Resonator-Enhanced Electro-Optic Frequency Comb Generators. *J. Light. Technol.* **38**, 1400–1413 (2020).

**Supplementary figures S1-S10 and table S1**

**
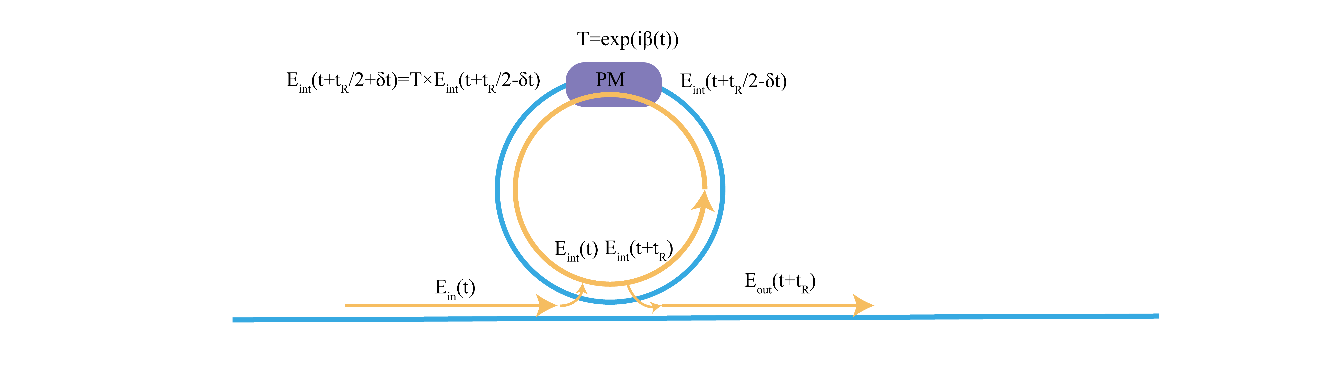
**

**Fig. S1 | Schematic diagram of cavity EO system.** We consider the entire cavity as a black box, where the Hamiltonian reflects the black box’s interaction with light. The black box receives the input light and releases it after one round trip. The PM contributes a transmission $T_{\mathrm{phase}}=e^{i\beta(t)}$. The Hamiltonian interaction occurs over a time $t_{R}$, which is not negligible.

**
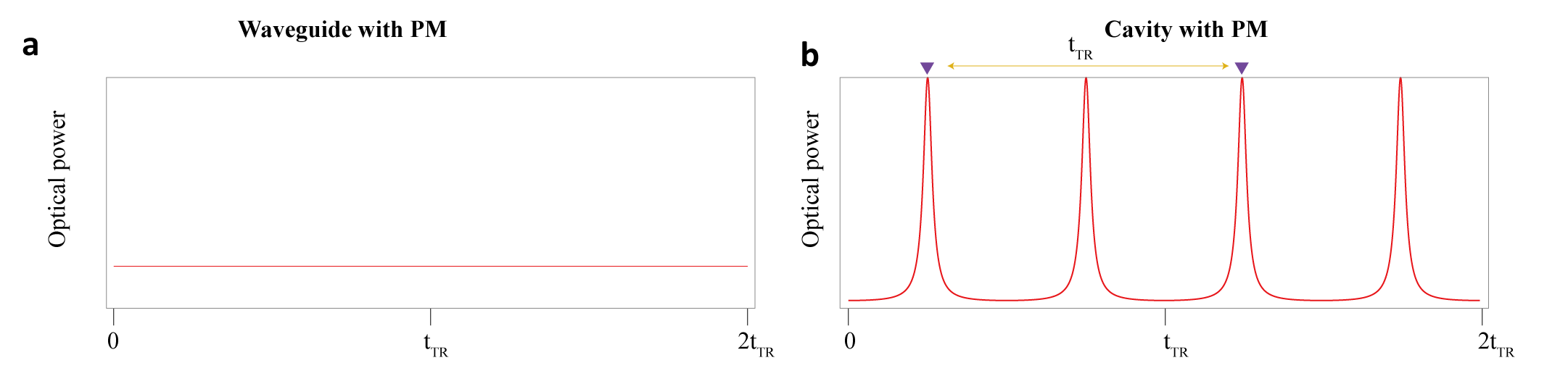
**

**Fig. S2| Difference between waveguide and cavity. a**, Output optical power by waveguide with PM. It remains unchanged because of PM not modulating the amplitude. **b**, Output optical power by cavity with PM. It exhibits EO pulses, leading to the discussion of roundtrip. This leads to the interaction Hamiltonian term $exp(i\beta(t))$

**
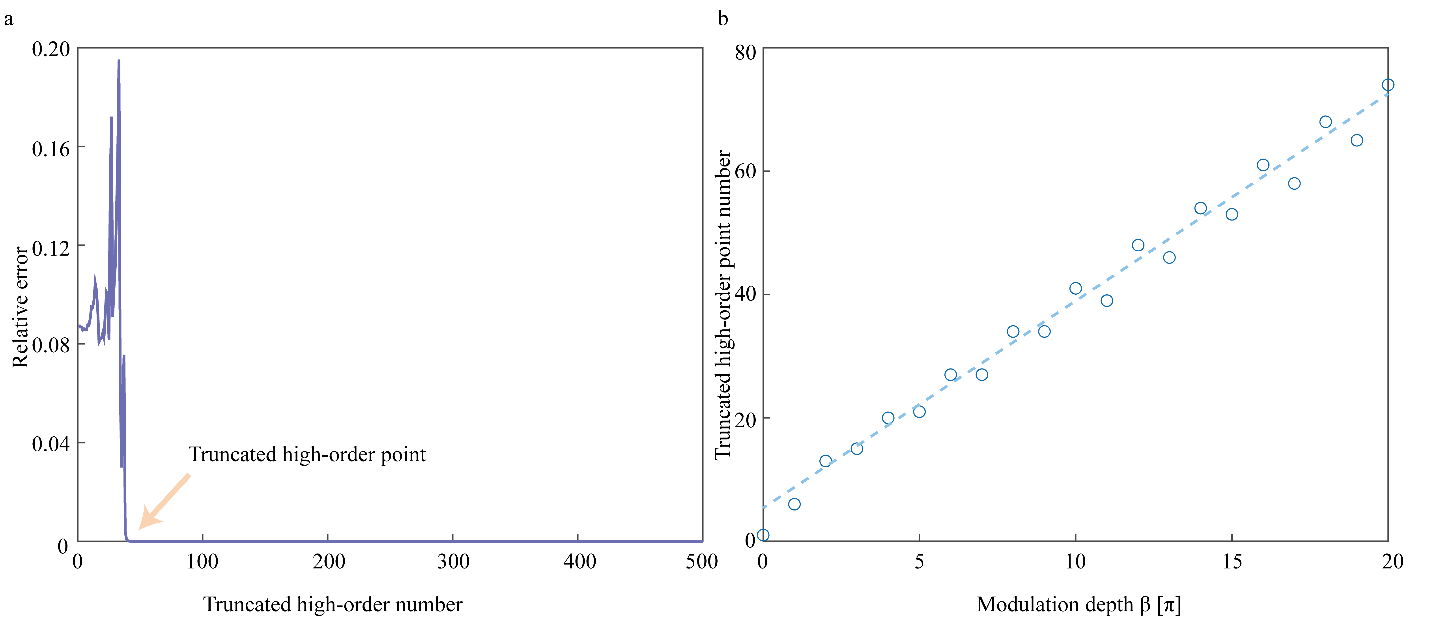
**

**Fig. S3 | Microwave high-order truncated number in coupled mode equation.** **a,** Numerical calculating error as a function of the truncated high-order number. Once the truncation exceeds a certain point (yellow arrow), increasing the truncated high-order number no longer reduces the error. **b,** The required truncation high-order point number for different modulation depths $\beta$. The point scales approximately linearly with the modulation depth $\beta$.

**
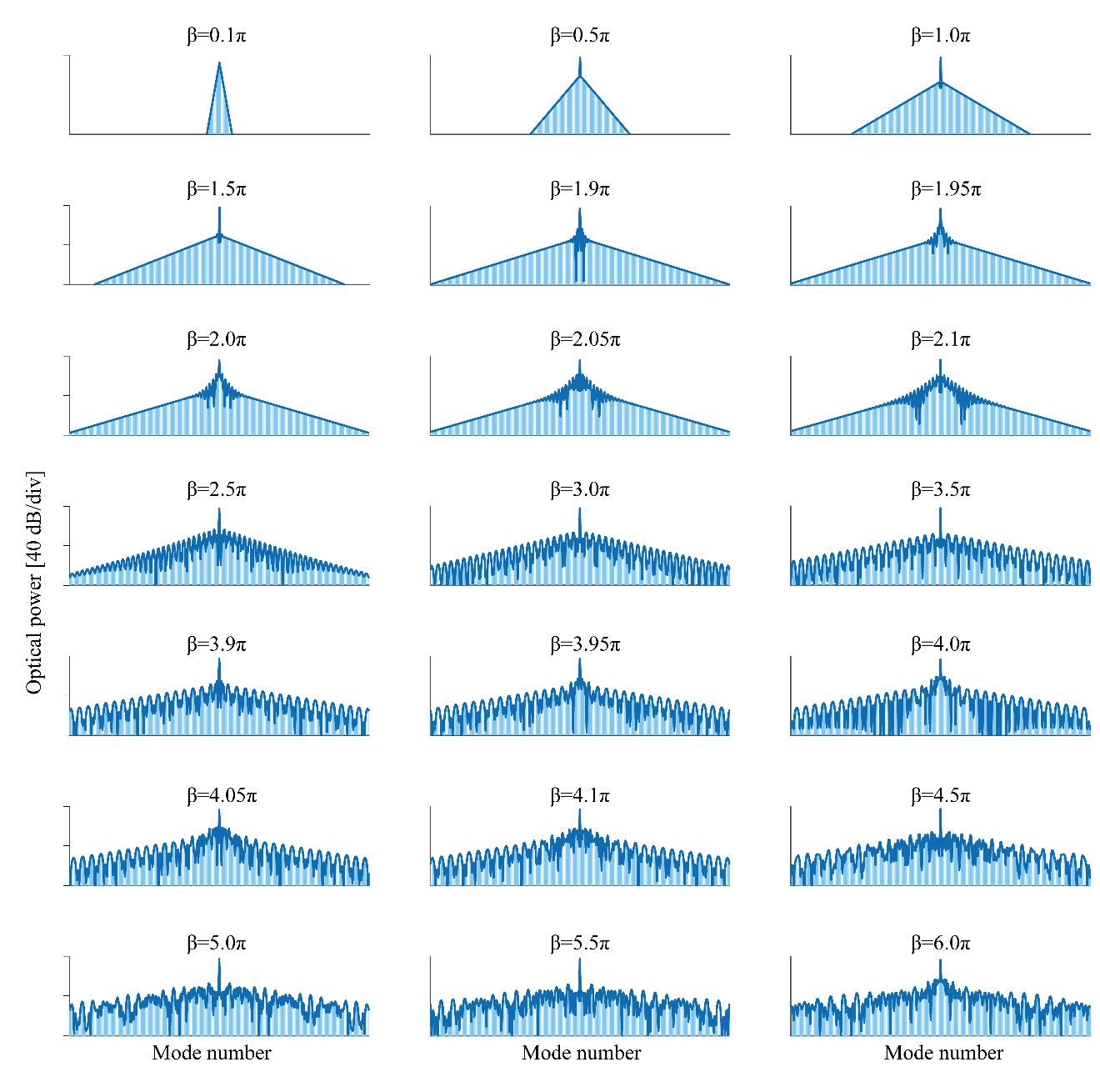
**

**Fig. S4 | Spectrum under zero pump detuning.** The spectra of different modulation strength $\Omega=\frac{\beta}{2\pi}\omega_{R}$ in zero detuning condition. The initial spectrum shows the triangle shape. We show the transition in detail around $\beta=2\pi$ and $\beta=4\pi$. The oscillation expands from the center around $\beta=2\pi$. The sub-peak exists around $\beta=4\pi$.

**
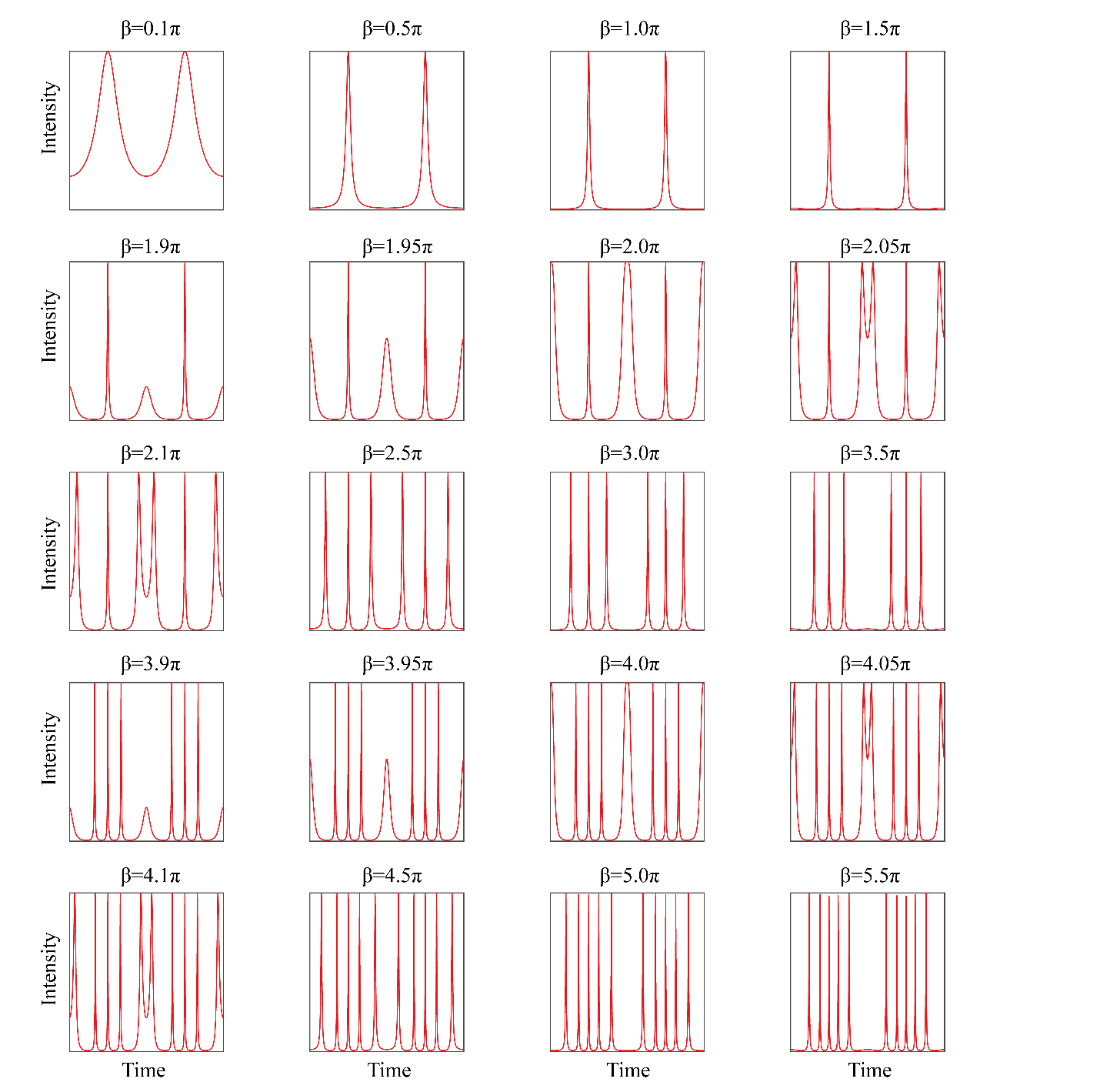
**

**Fig. S5 | EO pulses under zero pump detuning.** The time domain EO pulse of different modulation strength $\Omega=\frac{\beta}{2\pi}\omega_{R}$ in zero detuning condition. The new pulse develops and splits around $\beta=2\pi$ and $\beta=4\pi$. The pulse width narrower during the modulation strength increases.

**
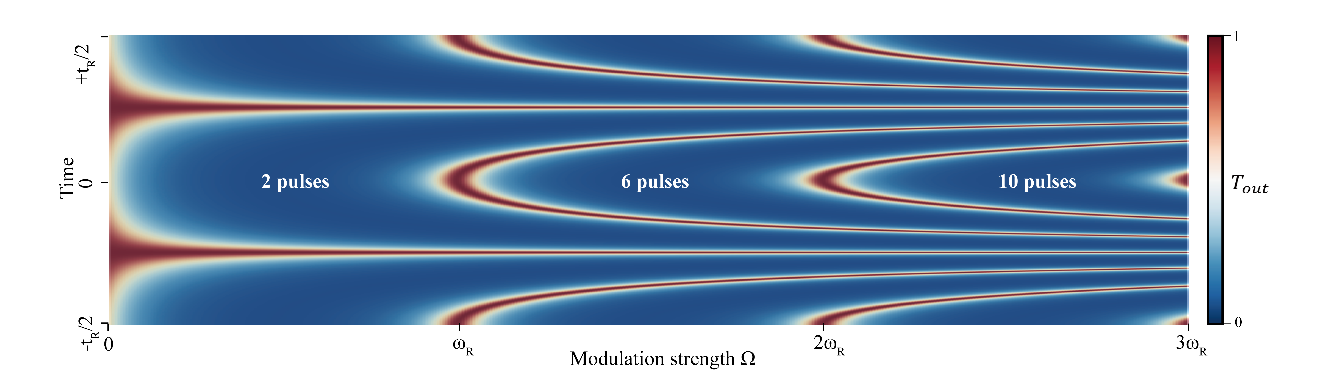
**

**Fig. S6 | The evolution diagram of EO pulses with modulation strength** $\boldsymbol{\Omega}$**.** As the modulation strength increases, the number of EO pulses inside the cavity undergoes a sudden change, forming the 2-pulses region, 6-pulses region, and 10-pulses region. Within each region, increasing the modulation strength $\Omega$ can reduce the pulse width, thereby broadening the spectrum. $T_{\mathrm{out}}$: transmission at the through port of the coupled waveguide.

**
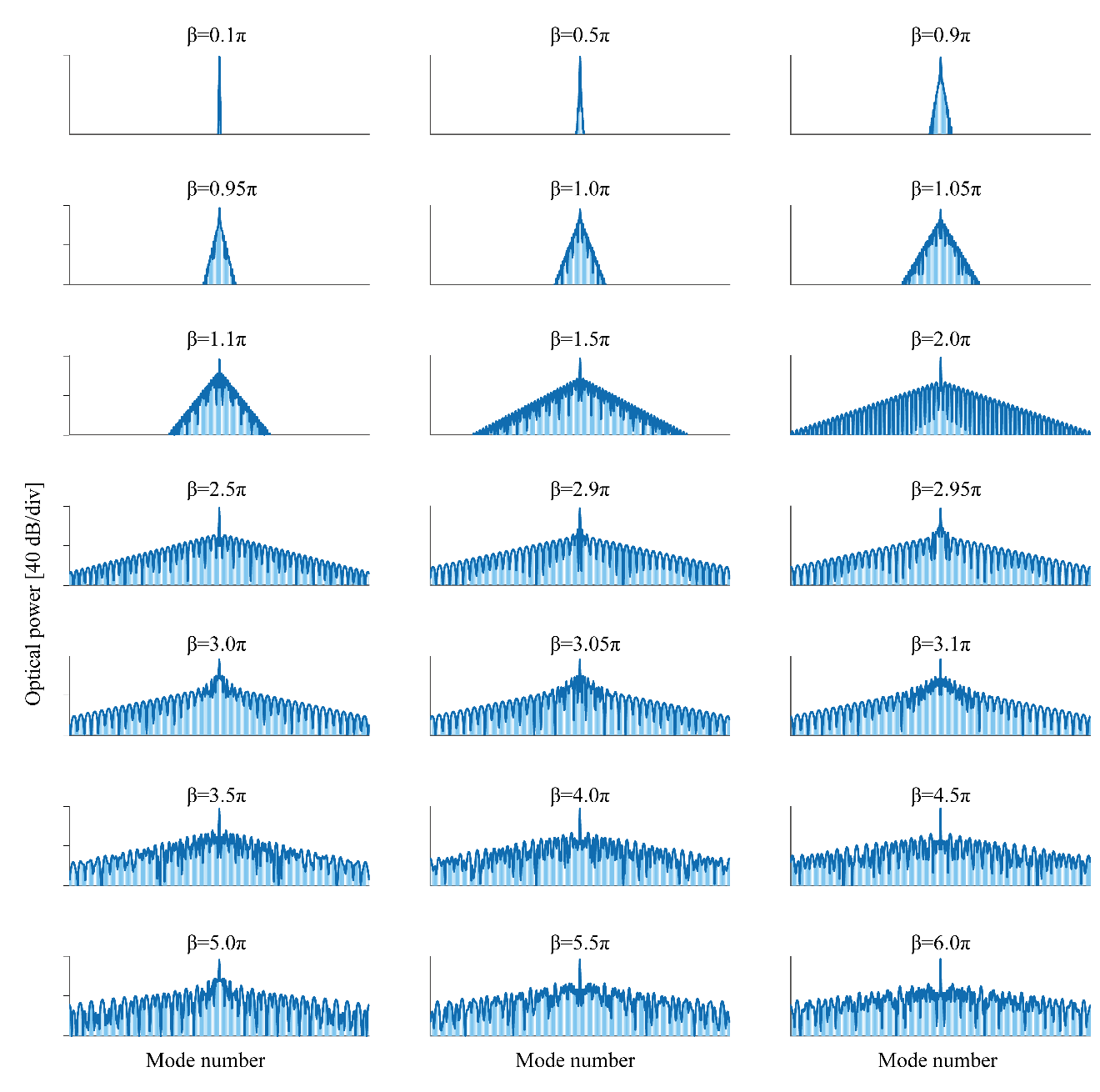
**

**Fig. S7 | Spectrum under maximum pump detuning.** The spectra of different modulation strength $\Omega=\frac{\beta}{2\pi}\omega_{R}$ in maximum detuning condition. The spectra experience the pump isolation and the similar shape in strong coupling region as the zero pump detuning condition.

**
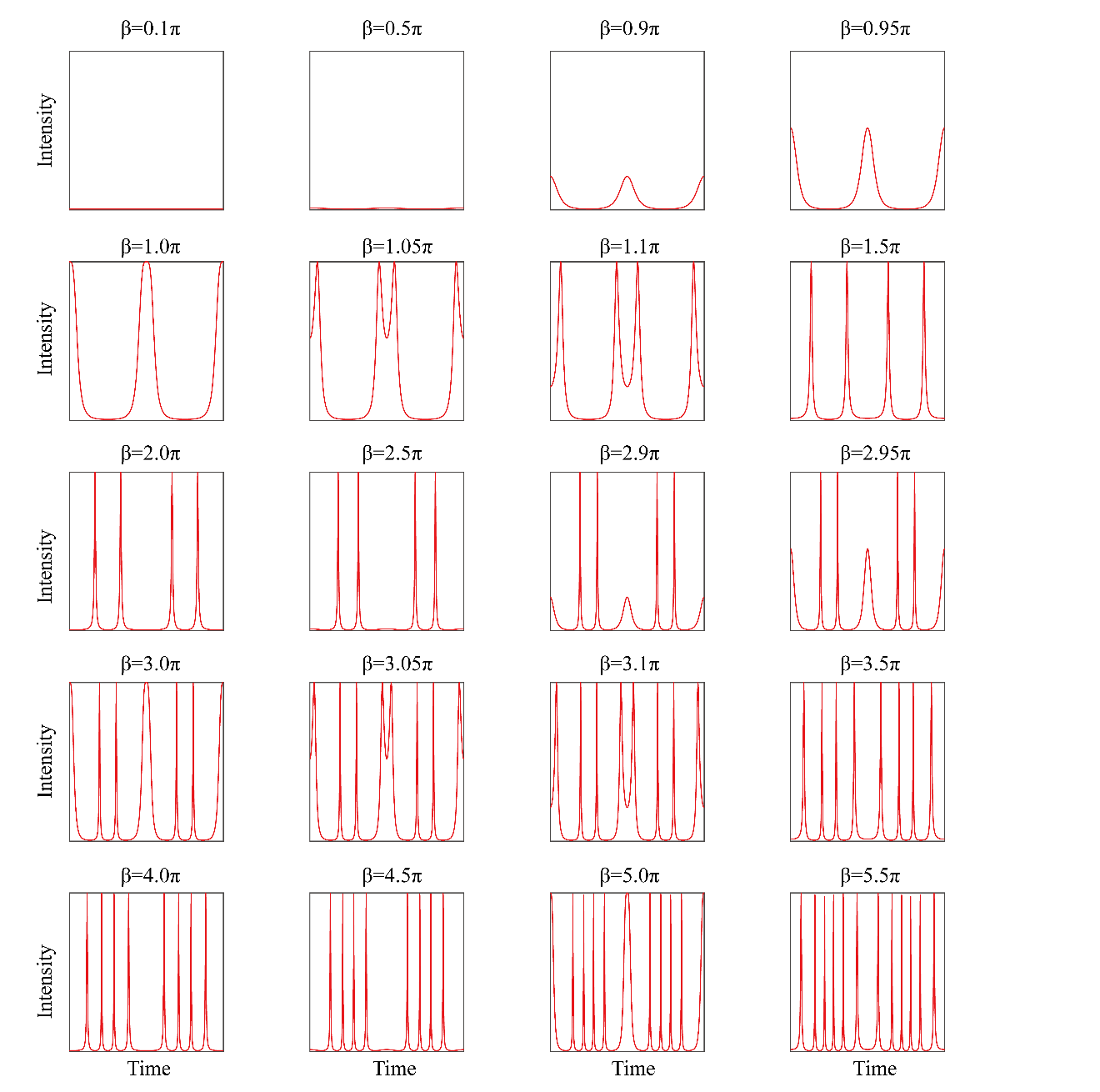
**

**Fig. S8 | EO pulses** **under maximum pump detuning.** The time domain EO pulse of different modulation strength $\Omega=\frac{\beta}{2\pi}\omega_{R}$ in maximum detuning condition. Different from the zero detuning condition, there exists 4 pulses instead of 6 pulses in strong coupling region and 8 pulses instead of 10 pulses in stronger coupling region.

**
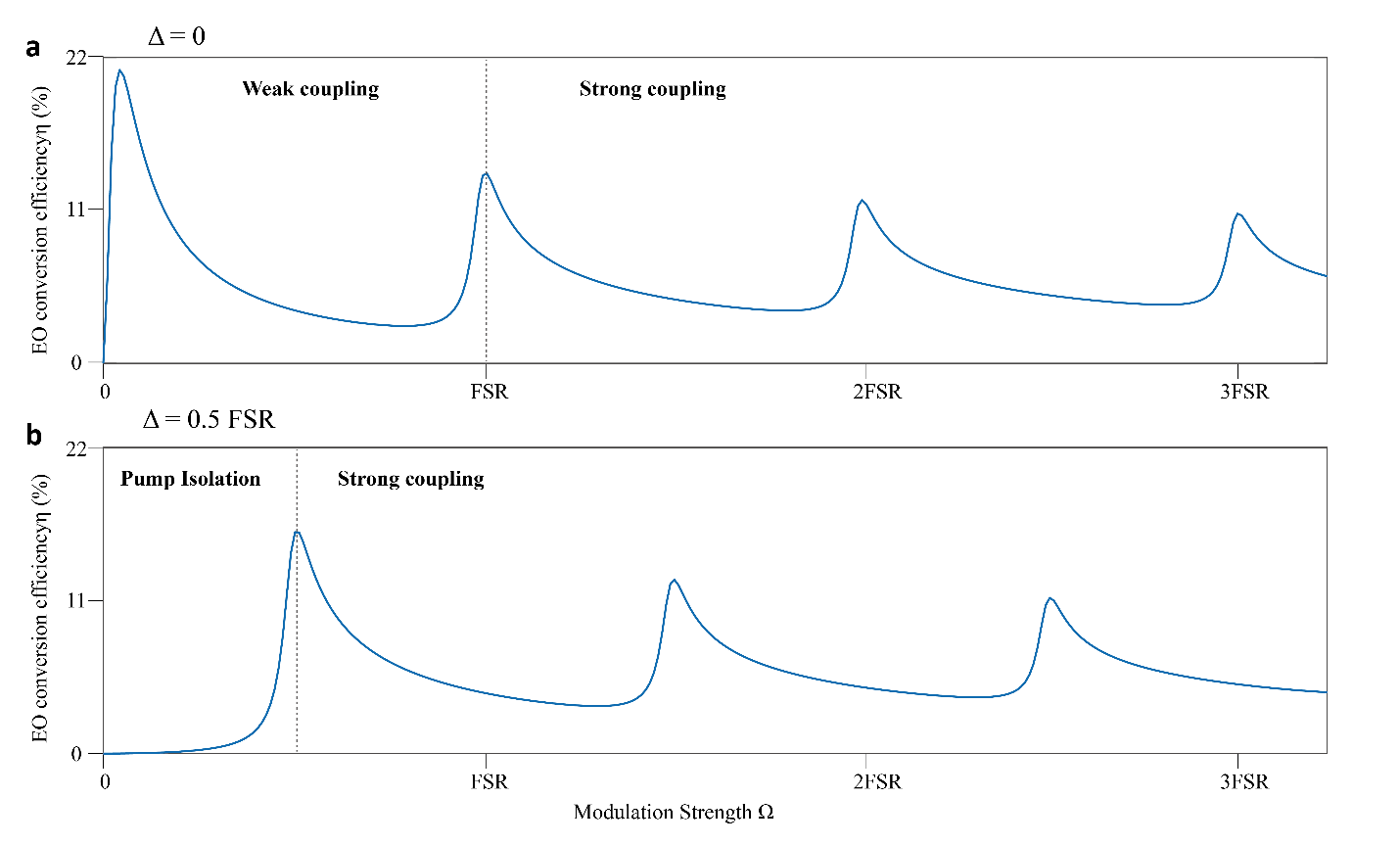
**

**Fig. S9 | EO conversion efficiency under zero and maximum pump detuning. a,** Zero detuning condition. There exists weak coupling region when $\Omega<\omega_{R}$ and strong coupling region when $\Omega>\omega_{R}$. The EO conversion efficiency increases and decreases periodically. **b,** Maximum detuning condition. There exists pump isolation region when $\Omega<\omega_{R}/2$ and strong coupling region when $\Omega>\omega_{R}/2$.

**
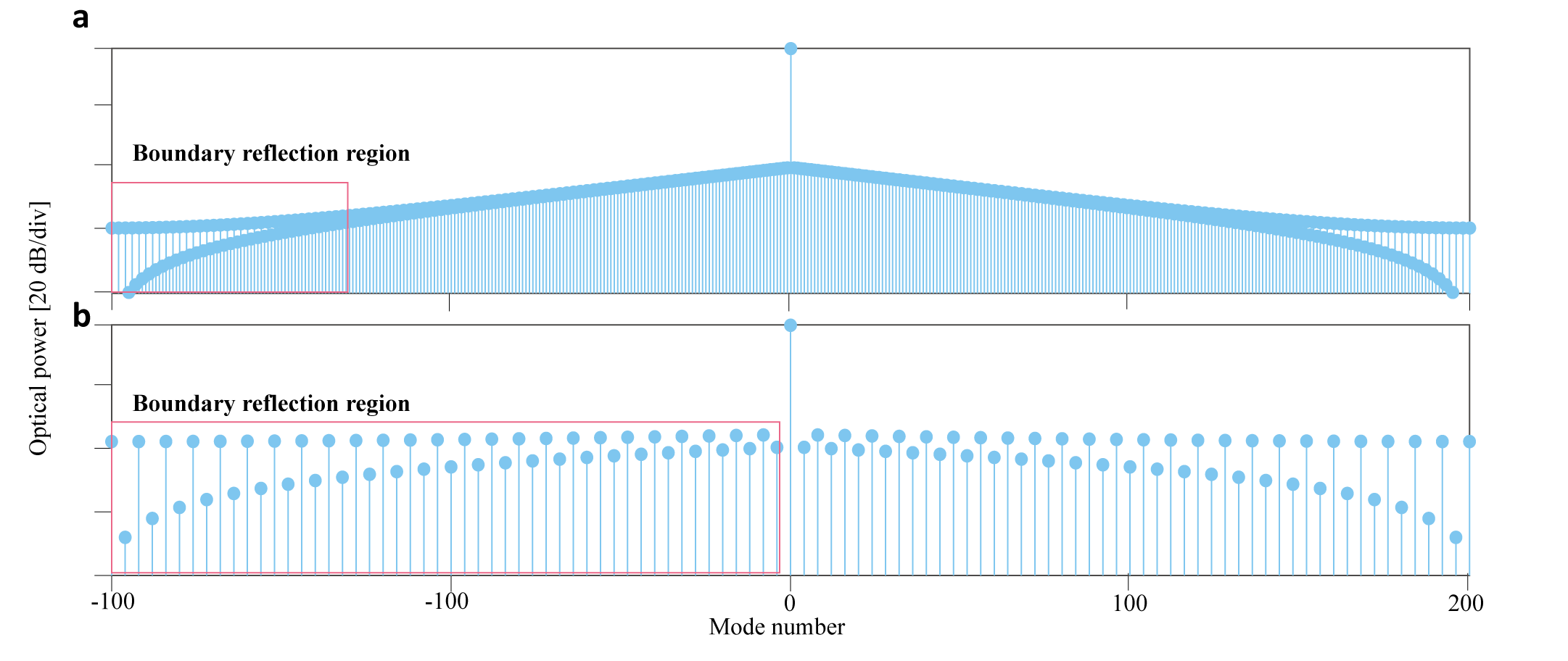
**

**Fig. S10 | The boundary reflection effect in different modulation frequency. a**, The boundary reflection effect when the modulation frequency equals to one FSR. In this case, fifty modes are influenced. The modes near the pump center are not influenced. **b**, The boundary reflection effect which modulation angle frequency equals to 4$\omega_{R}$. Almost all the modes are influenced, which means the entire spectrum is distorted by simulation. Increasing the total mode number can weaken this effect but at the expense of computation time when wider modulation bandwidths are considered.

**
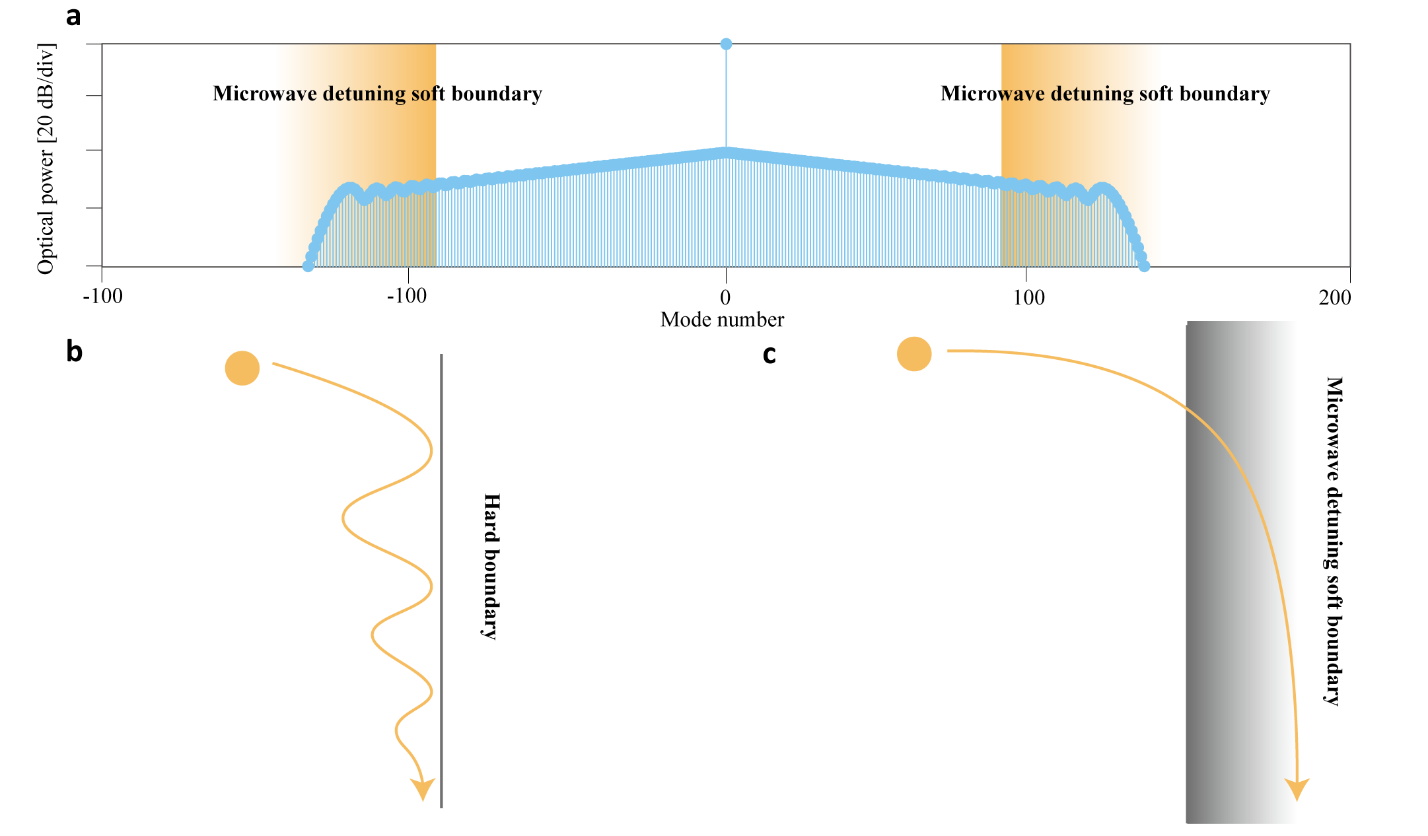
**

**Fig. S11 | The microwave detuning induced soft boundary. a**, Microwave detuning induced soft boundary. By utilizing the microwave detuning, we prevent the distortion near the pump center modes instead of simulation compensation methods without extra time and computing consumption. **b, c**, Hard boundary behaves as a wall and the pump energy propagation behaves as a falling ball. The ball bounces repeatedly when it hits a hard boundary, while the soft boundary can compensate for the severe oscillation.

**
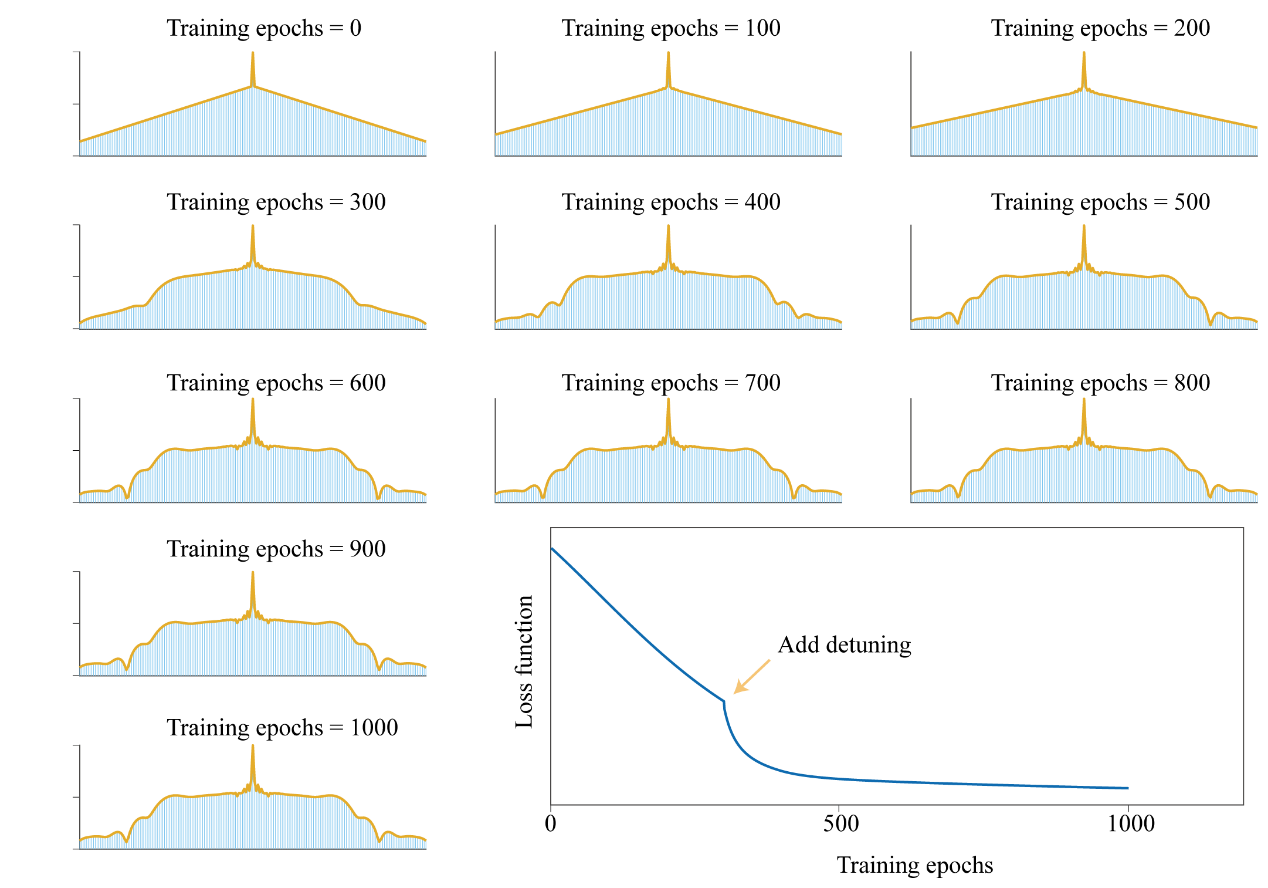
**

**Fig. S1****2 | Machine learning progress visualization.** Visualization of the machine learning progress in generating the flat-top EO comb. Adding microwave detuning can increase the learning speed in optimizing a flat-top EO comb.

**Table S1 | Machine learning trained modulation parameters**

| n | 1 | 2 | 3 | 4 | 5 | 6 | 7 | 8 | 9 |
| --- | --- | --- | --- | --- | --- | --- | --- | --- | --- |
| $\omega_{n}$(GHz) | 3.228 | 6.456 | 9.684 | 12.912 | 16.140 | 19.368 | 22.596 | 25.824 | 29.052 |
| $\beta_{n}$ | 4.8486 | 0.0151 | 0.2586 | 0.0090 | 0.3006 | 0.0057 | 0.3505 | 0.0020 | 0.4582 |
| $\phi_{n}$ | 1.549 | -3.060 | 1.332 | -1.919 | 1.621 | -1.825 | 1.799 | -2.144 | 1.748 |

**Supplementary videos 1-3**

**Video. 1 |** **Zero detuning EO spectra and pulse.** Gradually increasing the electro-optic modulation strength under pump-resonance condition without detuning, the linearity of the conventional electro-optic comb spectra (dB scale) is disrupted. Simultaneously, additional pulses appear in the time-domain signal corresponding to the Fourier transform. The left panel in the video: the electro-optic comb spectrum, the right panel: electro-optic time-domain pulses.

**Video. 2 | Maximum detuning EO spectra and pulse.** Gradually increasing the modulation strength under the condition of maximum pump detuning, unlike the non-detuned case in video 1, no electro-optic comb is observed at very weak modulation due to the severe pump detuning. However, when the modulation strength increases to modulation depth of $\beta=\pi$, the EO modulation demonstrates robustness against pump detuning for the electro-optic comb can still be generated despite the maximum pump detuning. Moreover, under this condition, the pump detuning exhibits entirely different frequency and time-domain dynamical characteristics compared to the non-detuned case. The left panel in the video: the electro-optic comb spectrum, the right panel: electro-optic time-domain pulses.

**Video. 3 | Visualization of ML generating flat-top EO comb.** Adding microwave detuning significantly enhances the flatness of the electro-optic comb during the machine-learning process. The left panel in the video: real-time electro-optic comb spectrum generated by machine learning, the right panel: loss function vs. training epochs.
